# Prolactin in relation to gestational diabetes and metabolic risk in pregnancy and postpartum: A systematic review and meta-analysis

**DOI:** 10.3389/fendo.2022.1069625

**Published:** 2022-12-22

**Authors:** Kate Rassie, Rinky Giri, Anju E. Joham, Aya Mousa, Helena Teede

**Affiliations:** ^1^ Monash Centre for Health Research and Implementation (MCHRI), School of Public Health and Preventive Medicine, Monash University, Melbourne, VIC, Australia; ^2^ Departments of Endocrinology and Diabetes, Monash Health, Melbourne, VIC, Australia

**Keywords:** pregnancy, prolactin, gestational diabetes mellitus, obesity, postpartum, lactation

## Abstract

**Context:**

Pre-clinical evidence suggests that prolactin has important metabolic functions in pregnancy and postpartum, in addition to lactogenic actions.

**Objective:**

To explore the relationship between prolactin and maternal metabolic outcomes in human pregnancy and postpartum, particularly in relation to gestational diabetes mellitus (GDM).

**Data sources:**

MEDLINE *via* OVID, CINAHL plus, Embase.

**Study selection:**

Eligible studies included women who were pregnant or up to 12 months postpartum, reporting at least one maternal serum prolactin level in relation to key metabolic outcomes including GDM, glycaemic parameters, obesity, and gestational weight gain.

**Data extraction:**

Two independent reviewers extracted data.

**Data synthesis:**

Twenty-six articles were included. Meta-analysis showed no relationship between maternal prolactin levels and GDM status, with a weighted mean difference of -2.14 ng/mL (95% CI -12.54 to 8.27 ng/mL, p=0.7) between GDM and controls in early pregnancy (n=3 studies) and -3.89 ng/mL (95% CI, -15.20 to 7.41 ng/mL, p=0.5) in late pregnancy (n=11 studies). In narrative synthesis of other outcomes (due to study heterogeneity and/or lack of data), prolactin levels were not associated with maternal glycaemic or weight-related parameters during pregnancy, but in the postpartum period (particularly with lactation) a high-prolactin environment was associated with low circulating insulin and beta-cell function, and increased insulin sensitivity.

**Conclusions:**

Current evidence from human studies does not clearly support a relationship between prolactin and metabolic parameters during pregnancy, including with GDM status. Elevated prolactin was associated with lower insulin and beta-cell function and higher insulin sensitivity in the post-partum period, but the direction of causality remains unclear.

**Systematic review registration:**

https://www.crd.york.ac.uk/prospero/, identifier [CRD42021262771].

## 1 Introduction

Human pregnancy is a period marked by profound reproductive and metabolic adaptations, including a progressive increase in maternal insulin resistance which is paralleled by increased maternal synthesis and secretion of insulin. Failure to sufficiently augment insulin secretion to overcome pregnancy-induced insulin resistance results in maternal gestational diabetes mellitus (GDM), defined as carbohydrate intolerance of variable severity with onset or first recognition during pregnancy (1). The resulting hyperglycaemia increases the risk for fetal macrosomia and obstetric complications (1, 2). An improved understanding of the mechanisms that drive changes in maternal insulin resistance, and further insights into biomarkers which can facilitate early identification of women at risk of GDM, are needed to optimise prevention efforts and mitigate potential complications.

Prolactin (PRL) is a 199-amino acid polypeptide hormone produced by lactotrophs in the anterior pituitary gland. It signals through the PRL receptor, with signal transduction activating the Janus kinase-2 signal transducer and activator of transcription 5 (JAK-STAT5) pathway. Whilst the hormone is best known for its lactogenic effect on the female mammary gland, PRL receptors are also found in tissues important in metabolism, such as pancreatic beta-cells, hepatocytes, adipocytes, macrophages, and skeletal muscle (3). PRL alters insulin sensitivity, adipocyte function and lipid metabolism *in vitro* in both human and animal models (4). During gestation, rising levels of lactogenic hormones such as PRL and placentally-derived human placental lactogen (hPL) may contribute to systemic insulin resistance and reduced insulin binding, but have also been directly implicated in the parallel process of maternal pancreatic beta-cell proliferation and increased insulin secretion. Such findings initially emerged predominantly from animal work (5), but have been corroborated by results from *in vitro* human studies (6, 7). As such, altered lactogen dynamics may contribute to the pathophysiology of insulin resistant conditions such as GDM (8, 9). Postpartum, observational evidence consistently links breastfeeding to improved long-term maternal metabolic outcomes, such as reduced risk of progression to type 2 diabetes mellitus (T2DM). Such benefits may be mediated, at least in part, by lactation-induced changes to carbohydrate and lipid metabolism and adipocyte biology (3), with PRL as a central hormonal regulator (10).

Narrative reviews (which constitute the majority of the existing work in this area, and have produced many of the current mechanistic hypotheses) are often incomplete or reach subjective conclusions, and all rely heavily on pre-clinical research, often conducted in animal models. Multiple observational studies over several decades have explored various aspects of the relationship between PRL and gestational metabolic outcomes in pregnant human populations, but have not yet been effectively synthesised.

In this systematic review, we examine current evidence regarding the relationship between PRL and maternal metabolic outcomes in pregnancy and postpartum, particularly in relation to GDM and maternal glycaemia, as well as GDM risk factors. We provide mechanistic insights and examine the clinical implications of these findings.

## 2 Systematic review question

In pregnant women (participants) what is the relationship between PRL levels (exposures) and:

(a) maternal gestational metabolic status/outcomes?
(b) maternal metabolic outcomes up to 12 months postpartum?

## 3 Methods

### 3.1 Protocol and registration

A protocol for this review has been previously published (11). The review is part of a larger evidence synthesis examining lactogenic hormones in pregnancy and postpartum, was conducted following the Preferred Reporting Items for Systematic Reviews and Meta-Analysis (PRISMA) guidelines, and is registered with the International Prospective Register of Systematic Reviews (PROSPERO), CRD42021262771.

### 3.2 Search strategy and databases

A systematic search strategy (**Supplementary Material 1**) combining MeSH terms and text words was developed using the OVID platform, in consultation with expert subject librarians, and was translated to other databases as appropriate. MEDLINE *via* OVID, MEDLINE ePub ahead of print, in-process, in-data review and other non-indexed citations *via* OVID, CINAHL plus, and Embase were searched from inception to 8 July 2021 (updated 9 May 2022).

### 3.3 Inclusion and exclusion Criteria

Selection criteria using a modified version of the Participant, Exposure, Comparison, Outcome and Study Type (PECOT) framework (11, 12), established *a priori*, were used to determine the eligibility of articles for inclusion in this review. Using this framework, studies were included when the following criteria were fulfilled: participants were pregnant women and women up to 12 months postpartum, regardless of lactation status and with any comparison group (or no comparison); endogenous maternal serum PRL must have been measured and reported at least once during pregnancy and/or up to 12 months postpartum; and at least one of the key maternal outcomes below were reported:

- GDM status during pregnancy, and diabetes status up to 12 months postpartum
- Metabolic indices (continuous measurements) related to maternal glucose/lipid metabolism (e.g. glucose measurements on oral glucose tolerance test; insulin secretion; insulin sensitivity/resistance indices; beta-cell function) during pregnancy or postpartum
- Body mass index/obesity, gestational weight gain
- Postpartum weight change
- Lipid profile

There were no date limits for eligibility, but only articles with full text available in English were included. Eligible study types included cross-sectional, longitudinal cohort or case-control, and randomised controlled trials. Narrative and systematic reviews were excluded, but their bibliographies were examined to identify relevant articles.

Key exclusion criteria included: populations with pathological PRL elevation (e.g. prolactinoma) in pregnancy; studies involving exogenous administration of PRL; studies involving an intervention or procedure to manipulate PRL; studies involving medications known to affect PRL (e.g. dopamine agonists); studies in which PRL was only measured in another fluid (e.g. amniotic fluid or cord blood); studies focused on assisted reproductive technologies or primarily focused on women with other pregnancy pathologies (e.g. pre-eclampsia, placental dysfunction, stillbirth); as well as animal studies and *in vitro*/tissue culture studies. Commentaries, letters, conference abstracts, and case reports were also excluded.

### 3.4 Study selection and risk of bias assessment

Two independent reviewers (KR and RG) screened all articles on abstracts and full text and assessed methodological quality of included studies, with 10% of quality assessments performed in duplicate. Quality appraisal (risk of bias) was performed on Covidence using the Monash Centre for Health Research and Implementation (MCHRI) Evidence Synthesis Program critical appraisal tool (**Supplementary Material 2**), which is based on the Newcastle‐Ottawa Scale for non‐randomised studies (13). Individual quality items were evaluated using a descriptive component approach to assess factors affecting external validity (methodology, inclusion/exclusion criteria, and appropriateness of measured outcomes) and internal validity (attrition, detection, selection and reporting bias, confounding, statistical analyses, and study power). Studies that fulfilled all, most or few criteria were deemed to have low, moderate, and high risk of bias, respectively. Disagreements at any stage were resolved through discussion between reviewers to reach a consensus.

### 3.5 Data extraction

Data were manually extracted from all included studies by two independent reviewers using a specifically developed data extraction form in Microsoft Excel. Duplicate extraction was performed for 10% of studies, with no discrepancies identified. Information was collected on general details (authors, reference/source, country, year of publication, study design, duration of follow-up), participants (baseline age, metabolic conditions, parity, body mass index [BMI], ethnicity, lactation status), PRL timepoints and values, PRL assay methodology, key maternal outcomes assessed in relation to PRL (unadjusted and adjusted, with consideration of covariates used), and conclusions.

### 3.6 Evidence synthesis and statistical analysis

Review Manager 5.4.1 software was used to perform meta-analysis for eligible outcomes. Where published papers contained insufficient data to be entered into meta-analysis, further details were sought from the authors. Random effects models were employed to generate weighted mean differences (WMD). Statistical heterogeneity was assessed using the I^2^ test, with I^2^ values of >50% indicating moderate to high heterogeneity. Sensitivity analyses were performed to explore the effects of studies with high risk of bias on the overall results. Recognising that older studies likely reflected a different clinical and research environment, sensitivity analysis was also performed with exclusion of studies published prior to 2000. For outcomes where meta-analysis was not possible (e.g. where studies were highly heterogeneous in methodology), narrative synthesis of results was performed. Data is presented in summary tables and in narrative format to describe the populations, exposures and key outcomes of the included studies. Forest plots and funnel plots have been used to present results from meta-analyses and publication bias assessments, respectively.

## 4 Results

### 4.1 Search results

**Figure 1:**
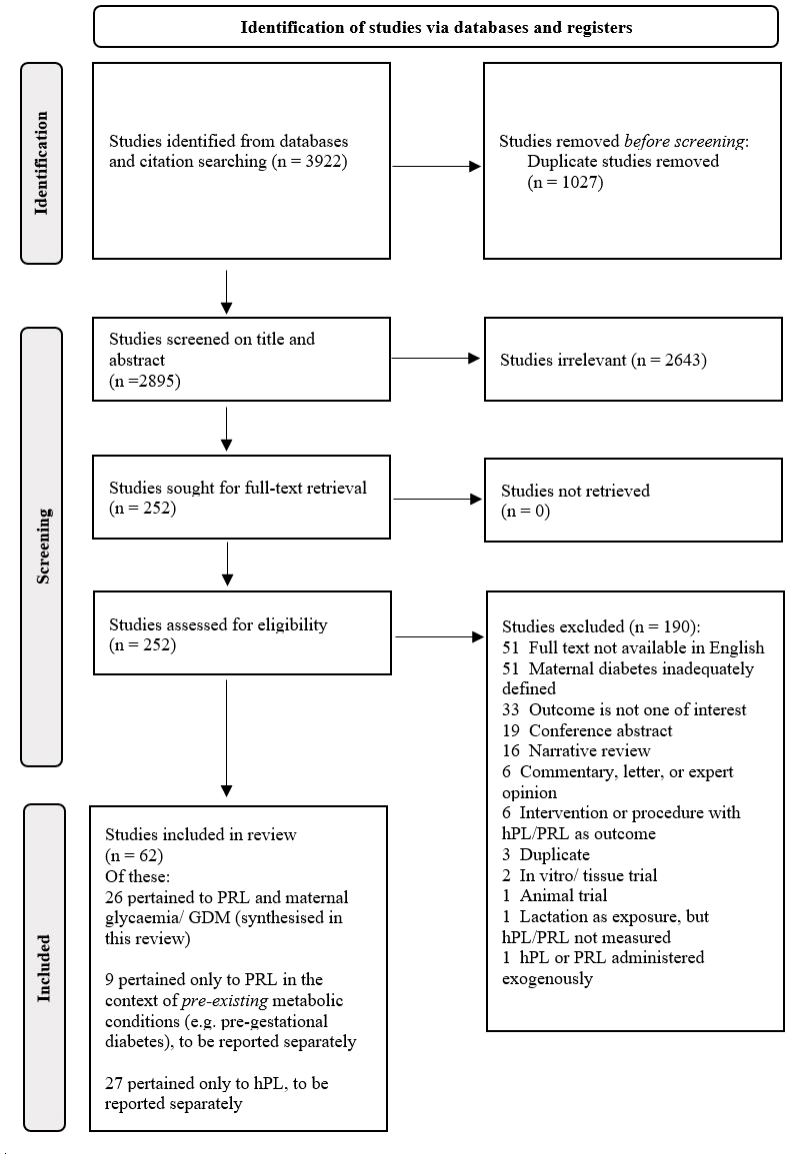
PRISMA flowchart.

A total of 3922 results were retrieved from the initial database search. Following removal of duplicates, 2643 and 190 studies were excluded at abstract and full text screening, respectively, with reasons documented for excluded full texts (**Figure 1**). Of note, the 51 studies excluded on the basis of English full text unavailability were disproportionately dated, with all but one published prior to 1997.

Of the 62 studies which met broader eligibility criteria for inclusion, 26 of these pertained to PRL in relation to maternal glycaemia (pregnancy/postpartum), GDM status, maternal weight or lipids, and were included in the present review. Meta-analysis was possible for exploring differences in early and late pregnancy PRL by GDM status, incorporating data from three and 11 studies for these timepoints, respectively (**Figures 2A**, **B**). All studies were observational in nature.

**Figure 2a:**
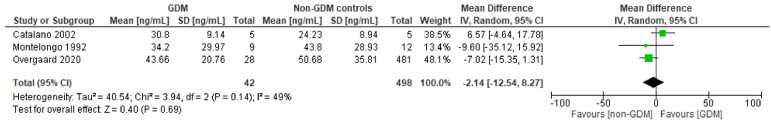
Forest plot showing meta-analysis of PRL levels in GDM vs non-GDM control women in early pregnancy (≤24 weeks) – 3 studies.

**Figure 2b:**
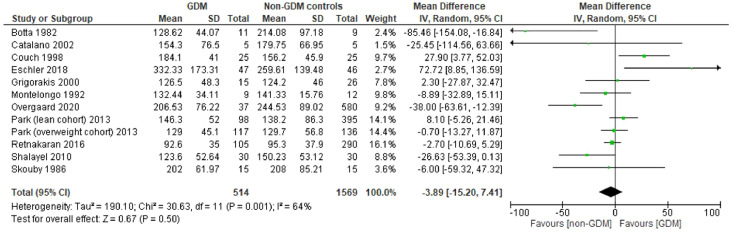
Forest plot showing meta-analysis of PRL levels in GDM vs non-GDM control women in late pregnancy (>24 weeks) – 11 studies.

### 4.2 Risk of bias and publication bias assessments

Of the 26 studies included, five were deemed high risk of bias, 15 moderate, and six low (**Tables 1**–**3**). The main aspects contributing to high risk of bias were statistical analysis (inadequate detail, inadequate description of methodology, lack of adjustment for key confounding variables), and variability in outcome measurement and reporting; which were present in four and three of the five studies deemed high risk of bias, respectively. Visual inspection of funnel plots was not suggestive of publication bias for any of the analyses (**Supplementary Material 3**).

**Table 1:** Studies examining PRL in relation to maternal GDM status, GDM risk, and/or continuous measures of maternal glycaemia in pregnancy - 15 studies.

Author and year	Design	Participants and sample size	Methodology	PRL pregnancy timepoints	Parameters analysed in relation to PRL	GDM definition used	Results	Authors’ conclusions	Risk of bias rating
**Botta et al, 1982** (14)	Cross sectional	n=11 GDM n=9 non-GDM controls	One-off PRL sampling at time of delivery	At delivery	GDM category	NDDG (OGTT in final week of preg)	Mean PRL sig. lower in GDM at delivery: GDM = 128.62 ± 44.07 vs. controls = 214.08 ± 97.18 ng/ml; sig.	PRL sig. lower in GDM than non-GDM women at time of delivery.	Moderate
**Catalano et al, 2002** (15)	Longitudinal observational	n=5 obese GDM n=5 non-GDM obese controls	PRL measured pre-conception, and then in early and late preg	Pre-conception 12-14 weeks 34-36 weeks	GDM category	Carpenter-Coustan	Mean PRL ( ± SEM) (ng/mL) difference NS between obese GDM and obese controls at any time point, with respective values: Pre-conception: 10.23 ± 3.62 vs 9.2 ± 3.81, NS At 12-14 weeks: 30.8 ± 6.9 vs 24.23 ± 4, NS At 34-36 weeks: 154.3 ± 34.23 vs 179.75 ± 29.94, NS	PRL NS different between obese GDM women and obese control women in either early or late preg.	High
**Couch et al, 1998** (16)	Longitudinal observational	n=25 GDM n=25 non-GDM controls	Three late-preg PRL measurements (after GDM diagnosis)	26-30 weeks 33-34 weeks 37-38 weeks	GDM category	NDDG	Mean PRL (ng/mL), overall and at each of the 3 time points was sig. higher in GDM than controls, with respective values: At 26-30 weeks: 124.8 ± 38.9 vs 114.1 ± 38.5, sig. At 33-34 weeks: 155.8 ± 48.5 vs 134.9 ± 45.0, sig. At 37-38 weeks: 184.1 ± 41.0 vs 156.2 ± 45.9, sig.	PRL higher in diet-controlled GDM than controls in late preg Higher PRL may contribute to higher triglycerides in GDM.	Low
**Ekinci et al, 2017** (17)	Longitudinal observational	n=69 women	OGTT for GDM screening at approx. 28 weeks. PRL measured at 35-39 weeks.	35-39 weeks	2h OGTT glucose	IADPSG	Sig. independent positive relationship between maternal PRL at 35-39 weeks and 2h OGTT result (adjusted for age, gravidity, parity and BMI). Each 1000 ng/mL increase in PRL was associated with a sig. median 0.34 nmol/L increase in 2h post-OGTT glucose (95% CI 0.01 - 0.69).	Higher third trimester PRL levels independently associated with reduced glucose tolerance as per 2h OGTT result, suggesting possible independent role for PRL in GDM pathogenesis.	High
**Eschler et al, 2018** (18)	Cross sectional	n=47 GDM n=46 non-GDM controls	One-off PRL sampling at 24-32 weeks	24-32 weeks	GDM category	Carpenter- Coustan	Mean PRL (ng/mL) difference NS between GDM and controls in late preg. At 24-32 weeks: GDM 332.33 ± 173.31 vs controls 259.61 ± 139.48, NS	PRL NS different between GDM women and controls in late preg.	Moderate
**Grigorakis et al, 2000** (19)	Cross sectional	n=15 GDM n=26 non-GDM matched controls	One-off PRL sampling at 28-32 weeks	28-32 weeks	GDM category	NDDG	Mean PRL (ng/mL) difference NS between GDM women and controls in late preg. At 28-32 weeks: GDM = 126.5 ± 48.3 vs controls 124.2 ± 46.0, NS	PRL NS different between GDM women and controls in late preg.	Moderate
**Kirwan et al, 2002** (20)	Longitudinal observational	n=5 GDM n=10 non-GDM controls (5 lean and 5 obese)	One-off PRL sampling at 34-36 weeks. Clamp studies pre-preg, at 10-12 weeks and 34-36 weeks	34-36 weeks	Insulin sensitivity	Carpenter-Coustan	Across whole cohort, no relationship between late preg PRL (at 34-36 wk) and insulin sensitivity by clamp at this time; (r=-0.13, p=0.67).	PRL in late preg not related to insulin sensitivity in this cohort, but may potentiate other circulating factors.	Moderate
**Li et al, 2020** (21)	Nested case-control	n=107 GDM cases n=214 non-GDM controls	PRL sampled at four points across gestation; OR of GDM using conditional logistic regression	10-14 weeks 15-26 weeks 23-31 weeks 33-39 weeks	GDM category GDM risk Glucose Insulin C-peptide HOMA-IR HbA1c, %	Carpenter-Coustan	Higher PRL in GDM than controls at 10–14 wk, median 50.4 vs. 42.1 ng/mL; sig. PRL difference NS between groups at 3 subsequent visits. At 10-14 wk, adjusted OR (95% CI) for GDM across increasing quartiles of PRL were 1.00, 1.13, 1.80 and 2.33; each 10 ng/mL increase of PRL associated with an OR=1.13 for GDM (95% CI [1.03, 1.25]). Similar results at 15-26 wk. Insulin and C-peptide: positively associated with PRL at 10-14wk (r= 0.25 and 0.23, respectively). NS association with insulin and inverse association with C-peptide at 15-26 wk. Glucose, HOMA-IR or HbA1c: NS association with PRL at either 10-14 or 15-26 wk. Note week 10-14 samples not fasted, week 15-26 fasted.	Prospective evidence of a positive association between early preg PRL levels and GDM risk. Suggested that higher PRL levels in early preg may be involved in GDM pathophysiology; before GDM diagnosis in latter half of preg.	Low
**Luthman et al, 1994** (22)	Cross sectional	n=12 GDM n=12 non-GDM controls	PRL sampling at baseline and during meal tolerance test in third trimester	29-38 weeks	GDM category	WHO 1980	PRL levels NS altered by breakfast meal, and NS diff between GDM and control women at all meal test timepoints.	PRL unaltered by ingestion of meal in third trimester of preg; and difference in GDM vs controls NS.	Moderate
**Montelongo et al, 1992** (23)	Longitudinal observational	n=9 early GDM, 7 insulin-treated n=12 healthy controls	PRL sampling in first, second and third trimesters (and postpartum, see **Table 3**)	9-10 weeks 21-23 weeks 32-34 weeks	GDM category	NDDG	Mean ( ± SEM) PRL (ng/mL) NS diff between GDM and controls at all timepoints (although trend to lower in GDM) At 9-10 wk: GDM = 34.20 ± 9.99 vs. controls = 43.80 ± 8.35; NS At 21-23 wk: GDM = 99.55 ± 18.03 vs. controls = 118.00 ± 7.69; NS At 32-34 wk: GDM = 132.44 ± 11.37 vs. controls = 141.33 ± 4.55; NS	Trend to lower PRL in GDM than control women across gestation, but difference NS at all time points. Increase of PRL across gestation parallels increase in lipoprotein Tg across gestation.	Moderate
**Overgaard et al, 2020** (24)	Longitudinal observational	n=37 GDM n=580 non-GDM controls	PRL sampling in early and late preg	Early preg, median 11.9 weeks (range 10.2-14.6) Late preg, median 29.0 weeks (range 28.5-29.5)	GDM category Early HbA1c Late HbA1c Late HOMA2-B Late HOMA2-IR	2h OGTT ≥ 9.0mmol/L	In early preg, PRL and PRL MoM NS different between women who developed GDM and those who remained NGT: median early preg PRL in GDM 40.5ng/mL vs. NGT 41.7; NS. In late preg, PRL and PRL MoM sig. lower in GDM vs NGT: median late preg PRL in GDM 200.6 ng/mL; NGT 238.4, sig. Each late preg PRL MoM increase was associated with GDM OR of 0.302 (95% CI 0.100, 0.913), p=0.034; adjusted on pre-preg BMI and maternal age. Late preg PRL ratio (T3PRL: T1PRL) difference NS between GDM and NGT groups, median GDM 5.05 vs NGT 5.91, NS. Early HbA1c: sig. neg assoc with early preg PRL, r= -0.193 Late HbA1c: sig. neg assoc with late preg PRL, r= -0.070 Late HOMA2-B sig. pos assoc with late preg PRL, r=0.053 Late HOMA2-IR NS assoc with late preg PRL	Low PRL levels in preg associated with higher HbA1c (in early and late preg) and higher GDM risk (in late preg). PRL was positively associated with markers of beta-cell function, but not IR.	Low
**Park et al, 2013** (25)	Cross sectional	n=215 GDM cases (98 lean, 117 overweight) n=531 non-GDM controls (395 lean, 136 overweight)	One-off PRL sampling at 24-28 weeks, analysed according to GDM and BMI category	24-28 weeks	GDM category BMI category (overweight defined as >23kg/m^2^)	Carpenter-Coustan	Mean PRL levels by group (ng/mL): lean non-GDM = 138.2 ± 86.3 lean GDM = 146.3 ± 52.0 overweight non-GDM = 129.7 ± 56.8 overweight GDM = 129.0 ± 45.1 PRL sig. diff between lean and overweight women (higher PRL in lean) but not by GDM status. PRL did not emerge as sig. predictor of either lean or overweight women’s GDM risk.	PRL lower in overweight Korean women regardless of GDM status. Overall, GDM in overweight Korean women linked to IR, in lean Korean women more likely mediated by insulin secretory deficit; PRL not likely implicated.	Low
**Retnakaran et al, 2016** (26)	Cross sectional	n=105 GDM n=290 non-GDM controls	One-off PRL sampling at time of OGTT, approx. 29 weeks	29 weeks	GDM category AUC glucose on OGTT Markers of insulin resistance Markers of insulin secretion	NDDG	Mean PRL NS different between GDM and non-GDM women: GDM = 92.6 ± 35 ng/mL vs. controls 95.3 ± 37.9 ng/mL, NS. Remained after adjustment for gestational week, maternal age, ethnicity, family history of DM, pre-preg BMI and GWG. AUC glucose on OGTT not associated with PRL in GDM women or in non-GDM women, including after adjustment. Matsuda index (whole body insulin sensitivity), HOMA-IR, fasting insulin: none sig. associated with PRL (in GDM or non-GDM women) after adjustment. ISSI-2 (beta-cell function), and insulinogenic index/HOMA-IR (beta-cell function): neither sig. associated with PRL (in GDM or non-GDM women) after adjustment.	PRL at time of OGTT NS different between GDM and non-GDM women, and not associated with AUC glucose. No association between PRL and markers of insulin resistance/sensitivity, or of beta-cell function. Circulating PRL may not be directly relevant to maternal glucose homeostasis.	Moderate
**Shalayel et al, 2010** (27)	Cross sectional	n=30 GDM* n=30 IGT* n=30 controls*	One-off PRL sampling in third trimester	Third trimester, not further defined	GDM category*	Non-pregnant WHO	Mean ( ± SEM) PRL levels by group (ng/mL) NS diff between groups: GDM* = 123.6 ± 9.61 vs IGT* = 144.3 ± 14.99 vs controls*= 150.23 ± 9.70, NS.	PRL NS different between GDM, IGT and control women in third trimester.	High
**Skouby et al, 1986** (28)	Longitudinal observational	n=15 GDM n=15 non-GDM controls	PRL sampling in third trimester, and at 4-8 weeks postpartum (see **Table 3** for latter)	33-38 weeks	GDM category Insulinogenic index	NDDG	Mean ( ± SEM) PRL levels (ng/mL) NS different between GDM women and controls at 33-38 weeks: GDM = 202 ± 16 vs controls 208 ± 22; NS Insulinogenic index NS associated with fasting PRL in either GDM or non-GDM women.	PRL NS different between GDM and control women in third trimester, and not related to insulinogenic index.	Moderate

GDM, gestational diabetes mellitus; DM, diabetes mellitus; PRL, prolactin; BMI, body mass index; sig., significant; NS, non-significant; OR, odds ratio; HOMA-IR, Homeostatic Model Assessment for Insulin Resistance; NDDG, National Diabetes Data Group; IADPSG, International Association of the Diabetes and Pregnancy Study Groups; NGT, normal glucose tolerance; MoM, multiple of the median; ISSI-2, insulin-secretion sensitivity index 2; AUC, area under the curve; IR, insulin resistance; GWG, gestational weight gain; SEM, standard error of the mean. Data are presented as mean ± SD unless otherwise specified in the table.

*Non-pregnant WHO diabetes criteria were used in this paper (despite the pregnant population), with “GDM” defined as fasting >7.8 or 2h >11.1 mmol/L, and “IGT” as fasting 6-7.8 or 2h 7.8-11.1 mmol/L. As such, the “GDM” group in this paper may have had overt diabetes, and the “IGT” group either GDM or overt diabetes; the “controls” may also have contained women with GDM according to appropriate pregnancy criteria.

**Table 2:** Studies examining PRL in relation to maternal BMI and/or gestational weight gain – 5 studies.

Author and year	Design	Participants and sample size	Methodology	PRL pregnancy timepoints	Metabolic parameters analysed in relation to PRL	Results	Authors’ conclusions	Risk of bias rating
**Lagiou et al, 2006** (29)	Longitudinal observational	n=270, Caucasian only subset of the cohort of Xu et al. (below); PGDM excluded	PRL measured at 16 weeks and 27 weeks	16 weeks 27 weeks	Maternal BMI pre-preg Maternal GWG	Maternal BMI pre-preg NS associated with either 16 or 27 wk PRL (after adjustment for key covariates). GWG NS associated with either 16 or 27 wk PRL (after adjustment for key covariates).	No relationship between PRL at 16 or 27 weeks and either maternal pre-preg BMI or GWG.	High
**Lappas et al, 2020** (30)	Cross sectional	n=69, all non-obese, none with PGDM or GDM	PRL measured at time of delivery	At time of elective Caesarean section	Maternal GWG	Excessive GWG group (n=35) had tendency to lower PRL than recommended GWG group (n=34): 159.5 ± 66.1 vs 194.0 ± 85.6 ng/mL, just NS (p=0.07). Est mean diff = -34.3 ng/mL, 95% CI (-71.3, 2.7). Each 1kg inc in GWG = -3.11 ng/mL PRL (95% CI -8.28, 2.07)	Trend to lower PRL levels in those with excess GWG than recommended GWG (at term), although just short of sig. PRL known to stimulate appetite in preg and involved in leptin resistance, so trend lower PRL in excess GWG group deemed ‘surprising’.	Moderate
**Park et al, 2013** (25)	Cross sectional	n=215 GDM cases (98 lean, 117 overweight) n=531 non-GDM controls (395 lean, 136 overweight)	One-off PRL sampling at 24-28 weeks	24-28 weeks	Maternal BMI category (overweight defined as >23kg/m^2^), with stratification by GDM status	Mean PRL levels by group (ng/mL): lean non-GDM = 138.2 ± 86.3 lean GDM = 146.3 ± 52.0 overweight non-GDM = 129.7 ± 56.8 overweight GDM = 129.0 ± 45.1 Sig diff between lean and overweight women (higher PRL in lean), but not between GDM/non-GDM groups.	PRL at 24-28 weeks lower in overweight Korean women regardless of GDM status.	Low
**Ren et al, 2022** (31)	Longitudinal observational	n=30 overweight/obese n=33 lean controls	PRL sampled at 37 weeks of preg, (and again at 48h postpartum, see **Table 3**)	37 weeks	Maternal pre-preg BMI category (overweight defined as >24 kg/m^2^)	Baseline PRL in ng/mL sig lower in overweight/obese women than lean control women at 37 weeks (overweight/obese = 231.80 ± 72.94 vs. lean = 304.29 ± 75.64; sig.)	PRL at 37 weeks lower in overweight/obese Chinese women than controls.	Low
**Xu et al, 2003** (32)	Longitudinal observational	n=304 Caucasian (USA) and n=335 Chinese; PGDM excluded	PRL measured at 16 weeks and 27 weeks	16 weeks 27 weeks	Maternal BMI pre-preg Maternal GWG, adjusted for pre-preg BMI	NS associated with PRL (both visits, adjusted for key covariates). NS associated with PRL (both visits, adjusted for key covariates).	NS association between PRL and adjusted GWG, or between PRL and maternal pre-preg BMI.	Moderate

PRL, prolactin; GDM, gestational diabetes mellitus; PGDM, pre-gestational diabetes mellitus; BMI, body mass index; GWG, gestational weight gain. Data are presented as mean ± SD unless otherwise specified in the table.

**Table 3:** Studies examining PRL in relation to maternal metabolism during lactation and postpartum – 10 studies.

Author and year	Design	Participants and sample size	Methodology	PRL postpartum timepoints	Metabolic parameters analysed in relation to PRL	Results	Authors’ conclusions	Risk of bias rating
**Erickson et al, 2020** (33)	Cross sectional	n=32 lactating mothers (all BMI <40, no PGDM or GDM)	On day 4-5 postpartum: PRL sampled at feed onset, and then at 20min	Day 4-5 postpartum	Maternal BMI at delivery	Maternal BMI at delivery NS related to baseline PRL at 4-5 days postpartum. Maternal BMI at delivery also NS related to PRL increase across feed.	No relationship between maternal BMI at delivery and either baseline PRL, or PRL increment across feed; at 4-5 days postpartum.	Moderate
**Harreiter et al, 2019** (34)	Cross sectional	n=106 n=51 had had GDM; n=11 had ongoing IGT 62 were lactating	One-off PRL measurement at time of OGTT at 3-5 months postpartum	3-5 months postpartum	Maternal pre-preg BMI Postpartum waist circ and hip circ, triglycerides (3-5mo PP), HDL, fasting glucose Total chol (3-5mo PP), LDL Fasting insulin, fasting C-peptide, HOMA-IR, pre- and post-hepatic beta-cell fx *Post glucose load:* OGIS, AUC insulin, IGT, GDM Insulinogenic index, Stumvoll 1st and 2^nd^ phase, disposition index, AUC ins/gluc AUC glucose	PRL neg assoc, -0.205, sig. PRL NS assoc PRL pos assoc, sig. PRL neg assoc, sig. PRL NS assoc. PRL neg assoc, sig. PRL pos assoc, sig.	Higher PRL levels assoc with lower pre-preg BMI and lower postpartum fasting insulin (univariate analyses, listed). After multivariate analysis, pre-hepatic beta-cell function and Stumvoll 1st phase insulin secretion index (but not BMI) independently and neg assoc with PRL levels. Conclusion was that beta-cell function lower in lactating than non-lactating women (independent of BMI) and is inv assoc with PRL. Both lean and obese lactating mothers have lower IR. Authors suggest that good beta-cell plasticity (allowing beta-cell function to fall from high insulin production during preg to low production postpartum) may enable PRL to rise with permissive effect on lactation.	Moderate
**Montelongo et al, 1992** (23)	Longitudinal observational	n=9 early GDM n=12 healthy controls	PRL sampled at 2-4 weeks postpartum (during lactation) and again after cessation of lactation	2-4 weeks postpartum, during lactation Post-lactation	Diabetes category	NS diff between mean ( ± SEM) PRL (ng/mL) either during lactation or post-lactation between GDM and controls At 2-4 wk postpartum (lactation): GDM 41.22 ± 10.71 vs. controls 62.54 ± 13.16; NS After cessation of lactation: GDM 7.33 ± 1.85 vs. controls 6.12 ± 0.83; NS	NS difference in PRL between GDM and control women in the postpartum period; either during lactation or after cessation thereof.	Moderate
**Nurek et al, 2021** (35)	Cross sectional	n= 20 healthy exclusively BF at 3mo postpartum n=17 healthy partially BF at 6mo postpartum n=17 healthy FF at 3-6mo postpartum	One-off fasting PRL sample	3-6 months postpartum	In lactating women only: Maternal BMI Maternal body weight Fasting insulin	NS PRL pos assoc, 0.281, sig NS	Lactation overall assoc with high basal PRL and low basal insulin levels (compared with non-lactating group). Within lactating group, PRL NS rel to insulin or maternal BMI. Pos assoc to absolute maternal weight found, but likely confounded by different measurement timing between partial and exclusively breastfeeding groups.	Moderate
**Ozisik et al, 2019** (36)	Cross sectional	n=12 lactating (2 had had GDM), n=11 non-lactating (none GDM)	One-off PRL measurement, and meal tolerance test	Postpartum period, not further defined	Across whole cohort: Hba1c 2 hour C-peptide HOMA-IR HOMA-IS AUC-insulin AUC-glucose In both lactating and non-lactating women: BMI Waist circumference Fasting glucose Fasting insulin Fasting C-peptide 1, 2, 3, 4, 5-hour glucose 1, 2, 3, 4, 5-hour insulin 1, 2, 3, 4, 5-hour C-peptide	PRL neg assoc, r= -0.564, sig. PRL neg assoc, r= -0.539, sig. PRL NS assoc PRL NS assoc PRL NS assoc PRL NS assoc PRL NS assoc PRL NS assoc PRL NS assoc PRL NS assoc PRL NS assoc PRL NS assoc PRL NS assoc PRL NS assoc	PRL in postpartum women (lactating and non-lactating) inversely associated with HbA1c and C-peptide. Authors state this supports “protective” effect of PRL in postpartum period and may reflect improved insulin sensitivity.	High
**Ramos-Roman et al, 2020** (37)	Cross sectional	n=12 lactating (83% exclusively breastfeeding), 8 had had GDM n=6 non-lactating (formula-feeding), 3 had had GDM	Extensive clinical studies, including hyperinsulinaemic euglycaemic clamp, at 5-8 weeks postpartum	5-8 weeks postpartum	For lactating women, during the clamp: R_a_ free fatty acid suppression % Free fatty acid suppression % Intrahepatic Tg% Plasma Tg	PRL neg assoc, r= -0.52, sig. PRL NS assoc, PRL neg assoc, r= -0.62, sig. PRL neg assoc, r = -0.57, sig.	Both lactating and non-lactating women had low insulin. Fasted, lactating women had 2.6x higher basal EGP and 2.3x rates of lipolysis compared with non-lactating. When hyperinsulinaemic euglycaemic clamp applied (mimicking fed state), both groups suppressed lipolysis and EGP, but lactating women needed 36% less insulin to do so, suggesting postpartum insulin sensitivity may be further augmented by lactation. In lactating group, higher PRL was related to better insulin-mediated suppression of lipolysis, and lower intra-hepatic and circulating Tg.	Low
**Rasmussen et al, 2014** (38)	Longitudinal observational	n=17 overweight/obese (BMI >26 kg/m^2^) n=23 lean controls	PRL measured at baseline, and 30min into breastfeeding: at 48h postpartum and then 7 days postpartum.	48 hours postpartum 7 days postpartum	Maternal pre-preg BMI category	Mean PRL response to suckling, ΔPRL (ng/mL) sig lower in overweight/obese women than lean control women at 48h, but not 7 days. At 48h: overweight/obese ΔPRL = -10.3 ± 28.3 vs. lean 26 ± 61.5; sig. At 7 days: overweight/obese ΔPRL = 57.1 ± 60.2 vs. lean 80.9 ± 67.6, NS	Women who were overweight or obese pre-conception had lower PRL response to suckling than normal-weight women at 48h, but not 7 days, postpartum. Maternal overweight/obesity was sig independent predictor of lower PRL response to suckling at both 48h and 7 days postpartum; which may explain higher rates of breastfeeding cessation in this group.	Moderate
**Ren et al, 2022** (31)	Longitudinal observational	n=30 overweight/obese (pre-preg BMI >24 kg/m^2^, Chinese pop) n=33 lean controls (pre-preg BMI 18.5-23.9 kg/m^2^)	PRL sampled at 37 weeks of preg, and again at 48h postpartum	37 weeks preg 48 hours postpartum	Maternal pre-preg BMI category	Baseline PRL in ng/mL sig lower in overweight/obese women than lean control women at both 37 weeks of preg (see **Table 2**) and 48h postpartum. At 48h postpartum overweight/obese 281.79 ± 87.61 vs. lean 392.96 ± 104.54, sig.	Women with pre-preg overweight/obesity had lower basal PRL levels at both 37 weeks preg and 48h postpartum than normal-weight peers. They also had significantly delayed onset of lactogenesis. Factors emerging as likely sig contributors to the lactogenesis delay (and poss also lower PRL levels) in the overweight/obese group were (a) higher late-preg leptin levels and (b) a slower fall in estrogen following delivery.	Low
**Retnakaran et al, 2016** (39)	Longitudinal observational	n=301 NGT n=60 pre-diabetes n=6 DM (based on OGTT at 3 months postpartum)	PRL sampled at time of OGTT in late second trimester of preg, but then analysed in relation to postpartum metabolic status	Late second trimester of preg (but related to postpartum metabolic status)	Maternal diabetes category at 3 mo postpartum Glycaemic markers at 3mo postpartum: Log Matsuda index Log HOMA-IR Log ISSI-2 Log IGI/HOMA-IR Fasting glucose AUC glucose Risk of pre-diabetes or DM at 3mo postpartum	Median (IQR) PRL in ng/mL had been sig higher in late 2^nd^ trimester in those with NGT than those with persistent dysglycaemia at 3 mo postpartum: NGT = 93.4 (72.9-121.9) vs. pre-diabetes 82.7 (60.4-97.5) vs. DM 79.2 (52.2-100.4); sig (adjusted for key postpartum variables) PRL NS assoc PRL NS assoc PRL pos assoc, 0.0016, sig. PRL pos assoc, 0.0031, sig. PRL NS assoc PRL neg assoc, -0.0111, sig. OR of pre DM or DM at 3mo PP for each SD increase of PRL in late preg: OR = 0.50 (0.35, 0.72), sig.(after adjusting for key postpartum variables)	PRL in late preg had been sig higher in those with NGT at 3mo postpartum than in those with postpartum pre-diabetes or DM. Higher late preg PRL independently predicted higher beta-cell function at 3 mo. PRL in late preg was independent predictor of risk of pre-DM or DM at 3mo PP (higher late preg PRL predicted lower dysglycaemia risk). Authors suggest that serum PRL in preg may provide novel insight into postpartum DM risk in young women, and suggest this might relate to known role of PRL in beta-cell mass expansion (here extending from preg into postpartum).	Moderate
**Skouby et al, 1986** (28)	Longitudinal observational	n=15 GDM, n=15 non-GDM all lactating at time of postpartum follow-up	PRL sampling and OGTT in late preg (33-38 weeks, see **Table 1**), repeated during lactation at 4-8 weeks postpartum	4-8 weeks postpartum	GDM status Change in OGTT glucose AUC between late preg and postpartum	Mean ( ± SEM) PRL during lactation NS different in GDM vs control women: GDM = 54 ± 9 vs controls 46 ± 10 ng/mL, NS. No relationship to change in PRL between late preg and postpartum (in either GDM or controls)	NS difference in mean PRL between GDM and non-GDM women either in later preg or at 4-8 weeks postpartum, during lactation. No assoc found between PRL change (preg to postpartum) and change in AUC glucose (preg to postpartum). PRL did not change during OGTT (either in preg or postpartum).	Moderate

BMI, body mass index; PGDM, pre-gestational diabetes mellitus; GDM, gestational diabetes mellitus; DM, diabetes mellitus; IGT, impaired glucose tolerance; NGT, normal glucose tolerance; OGTT, oral glucose tolerance test; PRL, prolactin; BF, breastfeeding; FF, formula feeding; HDL, high-density lipoprotein; LDL, low-density lipoprotein; HOMA-IR, Homeostatic Model Assessment for Insulin Resistance; AUC, area under the curve; NS, non significant; IR, insulin resistance; OGIS, oral glucose insulin sensitivity; Tg, triglycerides; OR, odds ratio; ISSI, insulin-secretion sensitivity index; IGI, insulinogenic index; R_a,_ rate of appearance; EGP, endogenous glucose production. Data are presented as mean ± SD unless otherwise specified in the table.

### 4.3 Prolactin in relation to GDM status and maternal glycaemic parameters in pregnancy

Fifteen studies (14–28) examined PRL in relation to GDM status (n=13 studies) and/or to maternal glycaemia in pregnancy (n=6 studies), with different time points for PRL measurements, as described below and in **Table 1**.

#### 4.3.1 Prolactin in GDM vs controls in early pregnancy (≤24 weeks)

Four studies (15, 21, 23, 24) measured PRL in the first trimester of pregnancy and correlated this to the subsequent development of GDM. Three of these (15, 23, 24) contained sufficient data for meta-analysis (**Figure 2A**), with pooled results suggesting no significant difference in early pregnancy PRL between GDM and non-GDM groups (WMD of -2.14 ng/mL, 95% CI -12.54 to 8.27 ng/mL, p=0.7) and moderate heterogeneity (I^2 =^ 49%, *p_het_* =0.1).

The fourth study (21) found significantly higher PRL in a multiracial cohort of GDM cases (n=104) compared with controls (n=213), representing the first large-scale prospective study of the association between early pregnancy PRL levels and GDM risk, and implicating PRL in the early pathophysiology of GDM. Results were reported in median and interquartile range format due to non-normal distributions for PRL, and – when contacted – the authors were unable to provide the original data for inclusion in the meta-analysis.

#### 4.3.2 Prolactin in GDM vs controls in late pregnancy (>24 weeks)

Thirteen studies compared PRL in late pregnancy between GDM and non-GDM pregnancies. Eleven of these contained sufficient data for meta-analysis, or this was supplied by the authors (**Figure 2B**) (14–16, 18, 19, 23–28). Where more than one late pregnancy timepoint was available in a study, the latest was used. Pooled data from these 11 studies showed no significant difference in late pregnancy PRL between women with GDM and controls (WMD of -3.89 ng/mL, 95% CI -15.20 to 7.41 ng/mL, p=0.5), with moderate heterogeneity (I^2^ = 64%, *p_het_* =0.001). Sensitivity analyses were performed stratifying by publication date (removing studies prior to vs after 2000) and risk of bias (removing studies deemed high risk of bias); with no major changes to overall effect or to heterogeneity.

A single study (24) examined the ratio of third trimester (29 week) to first trimester (12 week) PRL and compared this between GDM and non-GDM groups, finding no significant difference therein.

#### 4.3.3 Prolactin in relation to maternal glycaemic parameters in pregnancy

Maternal glucose measurements or oral glucose tolerance test (OGTT) results were analysed in direct relation to PRL in three studies. Ekinci et al. (17) reported that 2 hour OGTT glucose values at 28 weeks were positively related to PRL at 35-39 weeks. In contrast, the remaining studies reported that PRL was not cross-sectionally associated with plasma glucose at 10-14 or 15-26 weeks (21) or with glucose area under the curve at 29 weeks (26).

Insulin and C-peptide were related to PRL in two studies. One found no relationship between fasting insulin and PRL at 29 weeks (26), while the other (21) reported that PRL correlated positively with maternal insulin and C-peptide at 10-14 weeks, but by 15-26 weeks, the relationship with insulin was attenuated and PRL was inversely related to C-peptide (likely due to differences in fasting status between the two timepoints (21)).

HbA1c was measured in relation to PRL in two studies (21, 24), with one (21) reporting no relationship at 10-14 or 15-26 weeks, and the other (24) reporting an inverse association in both early (12 weeks) and late pregnancy (29 weeks).

Three studies reported the relationship between PRL and homeostatic model assessment of insulin resistance (HOMA-IR): one in early pregnancy, two in late pregnancy. All found no significant relationship (21, 24, 26).

Markers of beta-cell function (derived from insulin and glucose measurements) were reported in relation to PRL in three studies. One (24) reported a positive relationship between HOMA of beta-cell function (HOMA-β) and PRL in late pregnancy. In contrast, PRL was not associated with the insulinogenic index at 33-38 weeks (28), or with two similar derived measures of maternal beta-cell function at 29 weeks (26), in two other studies.

Only one group used gold-standard clamp techniques to directly measure insulin sensitivity (at 34-36 weeks) in a small cohort, finding no relationship with maternal PRL (20).

### 4.4 Prolactin in relation to maternal BMI and/or gestational weight gain in pregnancy

Five studies (**Table 2**) analysed PRL in relation to maternal pre-pregnancy BMI and/or gestational weight gain (GWG). Two reported lower PRL at 24-28 weeks in Korean women classified as overweight (BMI >23 kg/m^2^) (25) and at 37 weeks in Chinese women classified as overweight/obese (BMI >24 kg/m^2^) (31) compared with lean controls. For GWG, Lappas et al. (30) found no significant relationship, but described a trend toward lower PRL at delivery among non-obese, non-diabetic women in whom GWG exceeded recommended thresholds (compared with women with GWG within recommended ranges). The remaining two studies found no significant relationships between maternal PRL (measured at 16 and 27 weeks) and either pre-pregnancy BMI or GWG across a combined cohort of Chinese and Caucasian American women (32) or in a subset of the Caucasian American women only (29), after adjustment for multiple covariates.

### 4.5 Prolactin in relation to lipid profile parameters in pregnancy

Three studies analysed PRL in relation to maternal lipid profiles during pregnancy. Both Montelongo et al. (23) and Couch et al. (16) collected serial samples across pregnancy and found that (across all samples) PRL was significantly positively correlated with lipoprotein triglycerides (across all lipoprotein classes); likely reflecting the parallel tendency of both parameters to increase with advancing gestation.

### 4.6 Prolactin in relation to maternal glycaemia/metabolism during lactation and postpartum

Ten studies examined maternal serum PRL in relation to maternal metabolism in the postpartum period and/or during lactation (**Table 3**). These studies were particularly heterogeneous in their aims and methodology, precluding meta-analysis.

#### 4.6.1 Postpartum prolactin in relation to maternal GDM status in pregnancy

Three studies examined postpartum PRL following pregnancies affected by GDM (at various timepoints between 2 weeks and 5 months postpartum, and in both lactating and non-lactating women). All three suggested no significant difference in maternal serum PRL according to GDM status (23, 28, 34).

#### 4.6.2 Postpartum prolactin in relation to maternal BMI

Six studies examined maternal postpartum PRL in relation to BMI or overweight/obesity, of which three found negative associations and three found no relationship. Rasmussen et al. (38) showed that women with preconception overweight or obesity had a lower PRL response to infant suckling than their lean counterparts at 48 hours postpartum, and that maternal overweight/obesity preconception was an independent predictor of lower PRL response to suckling at 48 hours and 7 days postpartum. Similarly, Harreiter et al. (34) reported that pre-pregnancy BMI in lactating and non-lactating women at 3-5 months postpartum was negatively associated with PRL. Ren et al. (31) found lower PRL levels at both 37 weeks’ gestation and 48 hours postpartum in women with overweight/obesity than lean controls; accompanied by significantly more delayed lactogenesis. In the three remaining studies, two reported that maternal BMI was not associated with serum PRL postpartum (regardless of lactation status (36), or in lactating women at 3-6 months postpartum (35)); the third found no relationship between BMI at delivery and either pre-feed PRL or PRL response to a feed at 4-5 days postpartum (33).

#### 4.6.3 Prolactin in relation to other aspects of maternal postpartum metabolism

Five studies examined PRL in relation to broader aspects of maternal postpartum metabolism, including continuous metabolic parameters.

Ozisik et al. (36) reported an inverse association between postpartum PRL and both HbA1c and C-peptide in a small cohort of women (n=22, 12 of whom were lactating). Harreiter et al. (34) studied 106 women (n=51 with a history of GDM, and 61 of whom were lactating) at 3-5 months postpartum. On univariate analysis, PRL at this time was negatively associated with maternal pre-pregnancy BMI, fasting glucose, fasting insulin and C-peptide, HOMA-IR, and beta-cell function. On multivariate regression, pre-hepatic beta-cell function and first-phase insulin secretion remained independently and negatively associated with PRL. Of note, when women were stratified according to lactation status, this inverse relationship was only seen in the lactating women. In contrast, Nurek et al. (35) found no relationship between basal PRL and fasting insulin in 37 lactating women at 3-6 months postpartum.

Only one study (37) used hyperinsulinaemic euglycaemic clamp techniques to relate maternal glucoregulatory physiology postpartum to PRL, comparing lactating and non-lactating women. Circulating insulin levels were low in both groups. Lactating women had higher rates of endogenous glucose production and lipolysis during fasting than the non-lactating group. When the clamp was supplied (mimicking the fed state), lactating women required 36% less insulin for suppression of lipolysis compared with non-lactating women. Of note, *within* the lactating group, higher PRL levels were associated with better insulin-mediated suppression of lipolysis (as well as lower intrahepatic triglyceride content and lower circulating triglycerides).

A single study (39) related *pregnancy* PRL measurements to postpartum metabolic status. Here, a one-off measurement of PRL at 27-30 weeks was positively associated with subsequent maternal beta-cell function at 3 months postpartum, including after adjustment for lactation status.

## 5 Discussion

To our knowledge, this is the first systematic review to synthesise the evidence examining PRL in pregnancy and postpartum in relation to maternal metabolic and glycaemic outcomes, including GDM. Systematic reviews addressing mechanistic questions are relatively under-utilised in the endocrine literature, yet are key to assembling disparate data and setting future research agendas. Our results show no clear relationship between PRL and GDM status in the second half of pregnancy (following GDM development), but highlight the lack of evidence regarding the metabolic associations of PRL in early pregnancy. In the postpartum context, particularly with lactation, physiological PRL elevation is associated with low circulating insulin levels, low beta-cell function and insulin sensitivity; although the direction of causality remains unclear.

### 5.1 Prolactin in pregnancy metabolism and GDM aetiology

Pre-clinical evidence prior to this review provides strong theoretical support for the role of PRL in GDM aetiology. Hence, studies examining PRL in relation to GDM or maternal metabolic parameters during pregnancy comprised a large proportion of our review (13 of the 26 included studies). Among these studies, examination of PRL in early pregnancy (prior to GDM development) was a relatively uncommon approach. The largest of these studies prospectively linked higher first-trimester PRL levels to an increased risk of developing GDM (21), but this finding was not corroborated in the remaining studies (15, 23, 24). In studies focusing on PRL levels in late pregnancy (>24 weeks, at or after GDM development/diagnosis), our pooled meta-analysis suggested no significant differences between late pregnancy PRL in women with GDM compared with controls. Our findings thus suggest that late pregnancy PRL is likely not associated with GDM, but the contribution of earlier PRL concentrations and/or temporal variations in PRL to the development of GDM remains unknown.

These findings are interesting, given the growing body of general evidence in support of metabolic actions for PRL. In non-pregnant populations, observational evidence suggests that the effects of circulating PRL concentrations on metabolism and glucose homeostasis may be concentration-dependent (21). Large, population-based observational studies in non-pregnant, middle aged adults consistently suggest that higher PRL levels within the normal physiological range may be protective against the development of T2DM (40–44), a finding confirmed in a recent systematic review (45) (but not demonstrated here in the context of GDM). However, in states of pathological hyperprolactinaemia (such as in patients with prolactinoma, or treated with anti-psychotic medications), PRL levels well in excess of the normal range have been repeatedly associated with adverse metabolic outcomes including hyperinsulinaemia, insulin resistance, impaired endothelial function, elevated inflammatory markers and increased body weight (46–48). Such outcomes may be ameliorated by treatment of the pathological PRL elevation (with bromocriptine, for example) (47, 49–51). The contribution of PRL elevation to the hormonally-mediated insulin resistance of pregnancy is debated, but may again be dose-dependent: whilst low levels of PRL have been shown to inhibit lipolysis, the higher concentrations associated with late gestation have traditionally been thought to contribute to progressive stimulation of lipolysis and reduced insulin sensitivity (3, 52).

Furthermore, other research, largely conducted in animal models or *in vitro*, also provides strong theoretical support for the parallel role of PRL (alongside that of hPL) in promoting maternal islet cell adaptation and proliferation, a key adaptation to the increased insulin requirements of human pregnancy (6, 53). The increase in maternal insulin secretion during pregnancy is paralleled by increasing expression of PRL receptors on maternal pancreatic beta-cells, which bind PRL as well as hPL (52). This concept was established in pre-clinical rodent models, which consistently demonstrated marked increases in beta-cell proliferation and survival, insulin gene expression, and glucose-induced insulin secretion in response to both hormones *in vitro* and *in vivo* (6, 53). Indeed, knockout mice specifically lacking PRL receptors on pancreatic beta-cells have normal glucose tolerance outside of pregnancy, but become progressively glucose intolerant with gestation due to corresponding failure of beta-cell proliferation – essentially, developing GDM (54, 55). The direct applicability of these animal models to human beta-cell adaptation in pregnancy remains uncertain, although autopsy studies confirm increased beta-cell mass in pregnant women (56) and *in vitro* evidence shows that PRL (and hPL) directly enhance insulin secretion from human islets (6).

The lack of a clear relationship between PRL and GDM status in our review (despite the plausible mechanisms, outlined above, that emerge from non-pregnant humans and/or animal models) highlights the likely increased complexity of the interplay between PRL and maternal metabolism in human gestation. The hormonal milieu of pregnancy is multifaceted and synergistic, and several changes independent of PRL may modify insulin secretion and oppose insulin action in peripheral tissues. Rising levels of placental growth hormone, maternal insulin-like growth factor-1 (IGF-1), progesterone, tumor necrosis factor alpha (TNF-α) and cortisol, and a reduction in adiponectin; are also major contributors to progressive insulin resistance in late gestation; such that measurements of a single hormone in isolation are inherently problematic. Moreover, circulating serum levels provide only a partial description of hormone actions: for instance, recent evidence suggests that certain PRL receptor polymorphisms may predict GDM risk, implying differences at a cellular receptor level which may be just as important as absolute circulating hormone concentrations (57).

### 5.2 Prolactin and maternal metabolism in lactation and the postpartum period

In the postpartum period, physiological hyperprolactinaemia is the key endocrine change responsible for the initiation and maintenance of lactation. Lactation is a unique metabolic state associated with an elevation of plasma free fatty acids, and with the mobilisation of lipids from diet and adipose stores to the breast for milk production. Observational evidence suggests that lactation is associated with maternal metabolic benefits, with consistent findings of lower rates of persistent postpartum dysglycaemia and progression to T2DM in women who breastfeed compared with those who do not (both in the general population (58) and following GDM pregnancy (59)). Plausibly, then, PRL — as the central ‘breastfeeding hormone’ — may link effective and sustained lactogenesis to improved maternal metabolic status postpartum. Whether this is primarily mediated by improved beta-cell function or reduced insulin resistance remains unclear, as there are putative biological mechanisms for both (3, 34, 39).

Furthermore, adverse maternal metabolic environments may also have detrimental impacts on lactation success: observational evidence clearly demonstrates that women with obesity and/or diabetes are at significantly increased risk of lactogenesis delay and persistent poor milk supply (60, 61). Some of the studies in our review attempted to link PRL levels postpartum to maternal GDM status, none showing significant relationships: however, maternal lactation status was inconsistent, sample sizes were small and PRL sampling methodology frequently failed to account for the complex and pulsatile PRL dynamics that occur during breastfeeding.

The key findings from our review, however, emerged from those studies that measured PRL as part of a broader examination of the unique endocrine and metabolic environment of lactation. The studies included represent a small subset of a larger body of literature addressing the impact of breastfeeding on maternal metabolism: only studies that measured PRL and then related it directly to a maternal metabolic variable met our criteria for inclusion. Broadly, results support the concept of lactation (under the control of PRL) as a metabolic environment characterised by low circulating insulin levels, increased insulin sensitivity and low beta-cell function (34, 36, 37). Increased glucose concentrations post glucose load in the presence of low circulating insulin levels in healthy lactating women ensure the availability of glucose for lactose synthesis in the breast, which is not an insulin-dependent process (34). As such, the onset of lactation requires significant changes to glucose metabolism, beginning with beta-cell mass contraction, decreased insulin secretion and reduced peripheral insulin resistance. Lactation is also characterised by increased rates of maternal lipolysis and endogenous glucose production in the fasting state (when dietary substrate is unavailable for milk production) (37). However, the endocrine milieu of lactation is complex; and so the direct hormonal contribution of PRL to these metabolic changes is difficult to ascertain. Furthermore, the directionality of the relationship is unclear: does lactation (under the chief control of PRL) mediate improved postpartum metabolic outcomes, directly contributing to reduced insulin resistance? Or do metabolically-healthy women find it easier to successfully breastfeed, becoming over-represented in the ‘lactation’ groups in non-randomised observational studies (and having higher PRL simply by virtue of their lactation success)?

Arguments in both directions exist. Clamp data has suggested that (even among lactating women) higher PRL values may enhance insulin-mediated suppression of lipolysis, and lower both intra-hepatic and circulating triglycerides (37). The authors of this work have also previously suggested that lactation may improve postpartum insulin sensitivity by mobilising lipid accumulated in liver and muscle into breastmilk, instead of redirecting lipids into already enlarged adipocytes (3). Conversely, Harreiter et al. (34) have suggested that good “beta-cell plasticity” (i.e. an adequate increase in beta-cell function during pregnancy and an effective immediate decrease postpartum) is necessary to allow PRL to rise postpartum, exerting a ‘permissive’ effect on lactation. Indeed, reduced basal PRL levels (31) and reduced PRL responses to infant suckling (38) have been demonstrated in women with overweight/obesity in the studies in our review, and could potentially explain the well-established clinical phenomenon of lactogenesis delay in these individuals. The results of our review suggest that the relationship between PRL secretion and maternal metabolism in the postpartum period is complex, and may be bidirectional, but further data from well-designed and appropriately controlled studies are needed to further clarify these relationships and their implications for maternal and offspring health.

## 6 Strengths and limitations

Our review has unique strengths. As mentioned, to our knowledge, it is the first to systematically synthesise the clinical evidence linking PRL to maternal metabolic outcomes in human pregnancy and postpartum. It addresses a unique, mechanistic question linking metabolic and reproductive aspects of women’s health; and has allowed us to reach evidence-based conclusions and identify areas for future research.

Limitations of the review process included restricting the search to published work and to the English language.

Limitations of the collated literature included the relatively small number of eligible studies and the marked heterogeneity therein, which precluded meta-analysis for most outcomes. Variable study quality was reflected in the risk of bias assessments (20 of 26 [77%] were deemed to have ‘moderate’ or ‘high’ risk of bias). Studies were all observational, with small participant cohorts. In GDM studies specifically, PRL was often only sampled at a single late-pregnancy timepoint (most commonly after the development of GDM) and then compared between GDM and control groups. PRL levels start rising from the beginning of pregnancy and increase rapidly across the latter half of pregnancy, but sampling of PRL within a broad gestational age bracket (eg. 24-28 weeks) was a common approach, often without subsequent adjustment for exact gestational age at the time of collection. Furthermore, PRL exhibits significant diurnal rhythms (both inside and outside of pregnancy) (62), but timing and conditions of collection were unclear and/or unstandardised in many studies. Finally, the diagnostic criteria used to define GDM was inconsistent between studies (see **Table 1**), reflecting differences in their era of publication and region of origin.

In postpartum studies, methodology failed to acknowledge the complex dynamics of postpartum PRL secretion. Many studies compared one-off measurements of PRL amongst women within broad postpartum timeframes (e.g. 3-5 months postpartum), without detailed description of lactation status and intensity, presence/absence of supplemental feeding, or careful timing of sample collection relative to a feed.

PRL assay methodology also varied according to study age, with older studies using radioimmunoassay techniques and newer studies favouring enzyme-linked immunoassays. Finally, the hormonal environment of pregnancy and postpartum is complex, and studies that focus on absolute serum levels of a single hormone inevitably overlook other factors such as local tissue availability, hormone synergy, and receptor polymorphisms.

## 7 Conclusion

In summary, our findings suggest that whilst many human observational studies have attempted to link PRL to GDM pathophysiology, the available evidence is methodologically diverse and conflicting. Overall, there was no clear relationship between maternal PRL levels in late pregnancy and GDM status, while relationships with early pregnancy PRL (preceding GDM development), have not been established and await further study. There were no clear associations between PRL and other maternal glycaemic or weight-related parameters. In the postpartum state, particularly in the context of lactation, a high PRL environment is associated with low circulating insulin levels, low beta-cell function and increased insulin sensitivity. The exact contribution of PRL to these metabolic adaptations remains unclear and warrants further exploration.

## Data availability statement

The original contributions presented in the study are included in the article/**Supplementary Material**. Further inquiries can be directed to the corresponding author.

## Author contributions

KR conceptualised and designed the protocol, with oversight from AJ, AM and HT. KR designed the search strategy, conducted the search and obtained full copies of studies. KR and RG conducted screening, data extraction and risk of bias assessments. KR tabulated data, interpreted results, and performed statistical analysis with assistance from AM. KR drafted the manuscript, which was reviewed and approved by RG, AJ, AM and HT. All authors contributed to the article and approved the submitted version.
