# Supplementary material for: Prolactin in relation to gestational diabetes and metabolic risk in pregnancy and postpartum: A systematic review and meta-analysis
Source: Front Endocrinol (Lausanne). 2022 Dec 22;13:1069625. doi: 10.3389/fendo.2022.1069625 (PMC9813437; doi:10.3389/fendo.2022.1069625)
Supplement: Supplementary file 1 [file Table_1.docx]

**Supplementary material 1: Search strategy**

Ovid MEDLINE(R) and Epub Ahead of Print, In-Process, In-Data-Review & Other Non-Indexed Citations, Daily and Versions(R) <1946 to current>

1 (pregnan* or gestation* or post?partum or post-partum or lactat* or breastfe*).ti,ab.

2 exp pregnancy/

3 postpartum period/

4 lactation/

5 Breast Feeding/

6 1 or 2 or 3 or 4 or 5

7 prolactin.ti,ab.

8 prolactin/

9 placenta* lactogen*.ti,ab.

10 placental lactogen/

11 somato-mammotropin.ti,ab.

12 somato?mammotropin.ti,ab.

13 7 or 8 or 9 or 10 or 11 or 12

14 ((pregnan* or gestation* or matern* or post?partum or post-partum or birth or f?etal or baby or infant* or newborn* or neonat*) adj1 weight*).ti,ab.

15 (placenta* adj1 weight*).ti,ab.

16 (placenta* adj1 mass*).ti,ab.

17 (diabet* or glucose or obes* or metabolic).ti,ab.

18 polycystic ovar*.ti,ab.

19 exp diabetes mellitus/

20 glucose intolerance/

21 exp diabetes, gestational/

22 obesity, maternal/

23 birth weight/

24 fetal weight/

25 polycystic ovary syndrome/

26 pregnancy outcome/

27 14 or 15 or 16 or 17 or 18 or 19 or 20 or 21 or 22 or 23 or 24 or 25 or 26

28 6 and 13 and 27

29 exp animals/ not humans.sh.

30 28 not 29

Embase Classic+Embase <1947 to current>

1 (pregnan* or gestation* or post?partum or post-partum or lactat* or breastfe*).ti,ab.

2 exp pregnancy/

3 lactation/

4 breast feeding/

5 1 or 2 or 3 or 4

6 prolactin.ti,ab.

7 prolactin/

8 placenta* lactogen*.ti,ab.

9 placenta lactogen/

10 somato-mammotropin.ti,ab.

11 somato?mammotropin.ti,ab.

12 6 or 7 or 8 or 9 or 10 or 11

13 ((pregnan* or gestation* or matern* or post?partum or post-partum or birth or f?etal or baby or infant* or newborn* or neonat*) adj1 weight*).ti,ab.

14 (placenta* adj1 weight*).ti,ab.

15 (placenta* adj1 mass*).ti,ab.

16 (diabet* or glucose or obes* or metabolic).ti,ab.

17 polycystic ovar*.ti,ab.

18 exp diabetes mellitus/

19 glucose intolerance/

20 pregnancy diabetes mellitus/

21 maternal obesity/

22 birth weight/

23 fetus weight/

24 ovary polycystic disease/

25 pregnancy outcome/

26 13 or 14 or 15 or 16 or 17 or 18 or 19 or 20 or 21 or 22 or 23 or 24 or 25

27 5 and 12 and 26

28 (exp animal/ or exp invertebrate/ or nonhuman/ or animal experiment/ or animal tissue/ or animal model/ or exp plant/ or exp fungus/) not (exp human/ or human tissue/)

29 27 not 28

CINAHL PLUS

S26 S6 AND S12 AND S25

S25 S13 OR S14 OR S15 OR S16 OR S17 OR S18 OR S19 OR S20 OR S21 OR S22 OR S23 OR S24

S24 (MH "Pregnancy Outcomes")

S23 (MH "Polycystic Ovary Syndrome")

S22 (MH "Fetal Weight")

S21 (MH "Birth Weight")

S20 (MH "Obesity, Maternal")

S19 (MH "Diabetes Mellitus, Gestational")

S18 (MH "Glucose Intolerance")

S17 (MH "Diabetes Mellitus+")

S16 polycystic ovar*

S15 diabet* or glucose or obes* or metabolic

S14 placenta* N1 (weight* OR mass*)

S13 (pregnan* or gestation* or matern* or post?partum or postpartum or birth or f?etal or baby or infant* or newborn* or neonat*) N1 weight*

S12 S7 OR S8 OR S9 OR S10 OR S11

S11 somatomammotropin

S10 (MH "Placental Hormones")

S9 "placenta* lactogen*"

S8 (MH "Prolactin")

S7 prolactin

S6 S1 OR S2 OR S3 OR S4 OR S5

S5 (MH "Breast Feeding")

S4 (MH "Lactation")

S3 (MH "Postnatal Period+")

S2 (MH "Pregnancy+")

S1 pregnan* or gestation* or post?partum or postpartum or lactat* or breastfe*
