# Supplementary material for: Prolactin in relation to gestational diabetes and metabolic risk in pregnancy and postpartum: A systematic review and meta-analysis
Source: Front Endocrinol (Lausanne). 2022 Dec 22;13:1069625. doi: 10.3389/fendo.2022.1069625 (PMC9813437; doi:10.3389/fendo.2022.1069625)
Supplement: Supplementary file 2 [file Table_2.docx]

**Supplementary data 2: MCHRI critical appraisal template for assessment of risk of bias**

| **Study ID** | |  | | |
| --- | --- | --- | --- | --- |
| **Study citation** | |  | | |
| **EXTERNAL VALIDITY – IS THIS STUDY AND ITS RESULTS GENERALIZABLE TO MY SYSTEMATIC REVIEW QUESTION?** | | | | |
| **Patient/population/ participants** | | | Describe whether they were gender specific, had a particular condition or the general population, age and any other relevant characteristics (e.g. BMI) | |
| **Control population  (if appropriate)** | | | Describe whether they were gender specific, had a particular condition or the general population, age and any other relevant characteristics (e.g. BMI) | |
| **N** | | | Where possible, list the number of participants that were:   - Screened - Enrolled - Allocated/randomised - Assessed - Followed up | |
| **Setting** | | | List where the intervention was conducted and assessed ie. hospital, clinic, community and/or university setting. | |
| **Intervention/indicator** | | | Describe the intervention in as much detail as possible e.g. medication type, dose, duration, intervals. | |
| **Comparison/control** | | | Describe the comparison in as much detail as possible e.g. medication type, dose, duration, intervals. | |
| **Outcomes** | | | List what the study measured (e.g. weight, BMI, HbA1c) as primary outcomes and secondary outcomes. If the outcomes are not relevant to your systematic review, list these as measured but not relevant to your systematic review. | |
| **Does the study have a clearly focused question and/or PICO?** | | | Yes  Partial  No  Not reported | Consider if the question is ‘focused’ in terms of:  – the population studied  – the intervention given or exposure  - the comparison(s)  – the outcomes considered |
| **Inclusion Criteria** | | | Yes  No  Not reported |  |
| **Exclusion Criteria** | | | Yes  No  Not reported |  |
| **If there were specified inclusion/ exclusion criteria, were these appropriate?** | | | Yes  Partial  No  N/A | Consider if:  - the eligibility criteria used to specify the patients, interventions/ exposures and outcomes of interest. |
| **Is a cohort study the appropriate design to answer this question?** | | | Yes  Partial  No | Consider if a cohort study is a good way of answering the question under the circumstances. |
| **Were the outcomes measured appropriate?** | | | Yes  Partial  No  Not reported | Consider if the outcomes measured are appropriate and important outcome. |
| **Was there sufficient duration of follow-up for outcomes to occur?** | | | Yes  Partial  No  Not reported | May need to check with clinicians regarding what is sufficient duration for important events to occur.  An acceptable length of time should be decided before quality/risk of bias assessment begins. |
| **INTERNAL VALIDITY – HAS THIS STUDY BEEN CONDUCTED RIGOROUSLY IN ORDER TO REDUCE BIAS?** | | | | |
| **SELECTION BIAS** | **Other than the exposure under investigation, were the groups selected from similar populations?** | | Yes  Partial  No  Not reported | Consider:  - whether the different sources would affect outcomes e.g. one group recruited from hospital(s) the other from the community.  - time periods i.e. historical cohort  - whether there is a large difference in participation rate between the two arms of the study. |
|  | **Was the exposed cohort truly representative?** | | Yes  Partial  No  Not reported | This item is assessing the representativeness of exposed individuals in the community relevant to the study’s PICO, not the representativeness of the sample of individuals in the general population.  Consider:  - whether truly representative in the community (least bias)  - whether somewhat representative (some bias)  - whether selected group of users (bias)  - no description of the derivation of the cohort (most bias) |
|  | **Is it clear that the outcome of interest was not present at the start of study?** | | Yes  Partial  No  Not reported | In the case of mortality studies, outcome of interest is still the presence of a disease/ incident, rather than death. That is to say that a statement of no history of disease or incident is least biased. |
| **PERFORMANCE BIAS** | **Aside from the exposure, were the groups treated the same?** | | Yes  Partial  No  Not reported | To be sure it’s the exposure which is responsible for the effect. |
|  |  |  |  |  |
|  |  |  |  |  |
| **DETECTION BIAS** | **Was exposure measured in a standard, valid and reliable way?** | | Yes  Partial  No  Not reported | Where exposure measures require any degree of subjectivity, some evidence should be provided that the measures used are reliable and have been validated prior to their use in the study.  Consider whether ascertainment of exposure was determined by:  - secure record (eg surgical records) (least bias)  - structured interview  - written self report (bias)  - no description (most bias) |
|  | **Were outcome assessors blind to the exposure?** | | Yes  Partial  No  Not reported | Consider:  - If the outcome is objective (e.g. death) then blinding is less critical.  - If the outcome is subjective (e.g. symptoms or function) then blinding of the outcome assessor is critical. |
|  | **Were all outcomes measured in a standard, valid and reliable way?** | | Yes  Partial  No  Not reported | Where outcome measures require any degree of subjectivity, some evidence should be provided that the measures used are reliable and have been validated prior to their use in the study.  For some outcomes (e.g. fractured hip), reference to the medical record is sufficient to satisfy the requirement for confirmation of the fracture. This would not be adequate for vertebral fracture outcomes where reference to x-rays would be required.  Consider whether outcomes were determined through:  - independent blind assessment or confirmation of the outcome by reference to secure records (x-rays, medical records, etc.) (least bias)  - record linkage (e.g. identified through codes on database records)  - self report (i.e. no reference to original medical records or x-rays to confirm the outcome) (bias)  - no description (most bias) |
|  | **Were outcomes assessed objectively and independently?** | | Yes  Partial  No  Not reported | Independence of assessment is important where the result of one outcome may effect the interpretation of another.  When outcomes are objectively assessed, their independence from each other is less important. |
| **ATTRITION BIAS** | **What percentage of the individuals recruited into each arm of the study were lost to follow up?** | | X% treatment  X% control/ comparison  Not reported | Consider:  - if all patients who entered the trial were properly accounted for and attributed at its conclusion.  - why patients dropped out, as well as how many.  - the drop out rate may be expected to be higher in studies conducted over a long period of time.  - if comparisons were made between participants followed-up and those lost to follow up, by exposure status. |
|  | **What percentage of the individuals were not included in the analysis?** | | X% treatment  X% control/ comparison  Not reported | Consider:  - if analysis was as per protocol or intention to treat  - number of crossovers  - reason for crossover |
| **REPORT BIAS** | **Is the paper free of selective outcome reporting?** | | Yes  Partial  No  Not reported | Consider:  - if all the planned outcomes were measured  - if all the measured outcomes were reported  - if any additional or composite outcomes were measured.  This is difficult to determine if there isn’t a protocol. |
| **CONFOUNDING** | **Are the cohorts comparable on the basis of design or analysis?** | | Yes  Partial  No  Not reported | Consider  - either exposed and non-exposed individuals must be matched in the design and/or confounders must be adjusted for in the analysis.  - statements of no differences between groups or that differences were not statistically significant are not sufficient for establishing comparability.  Note: If the relative risk for the exposure of interest is adjusted for the confounders listed, then the groups will be considered to be comparable on each variable used in the adjustment. |
| **OTHER INTERNAL VALIDITY/BIAS** | **Were there any conflicts of interest in the writing or funding of this study?** | | Yes  No  Not reported | Consider:  - if any of the authors are/were employed, sponsored etc by pharmaceutical companies, or have other financial/other ties  - if any commercial companies were involved in funding, writing, editing, data analysis or manuscript approval |
|  | **Was the study sufficiently powered to detect any differences between the groups?** | | Yes  Partial  No  Not reported | Consider:  - if an adequate sample size calculation was undertaken  - if the required sample size recruited and retained  - for which outcomes the study was powered  - if confidence intervals include a clinically important difference, the study was underpowered  NB this is less important if significant differences were found. |
|  | **If statistical analysis was undertaken, was this appropriate?** | | Yes  Partial  No  Not reported  N/A | Consider:  - whether the authors performed any statistical tests or just presented figures  - if the statistical analysis was planned a priori  - if the data were analysed accordingly to the study protocol.  - the type of data and the statistical tests used. (Please refer to the CCE workbook as required)  - use of parametric versus non-parametric tests; whether the data has been checked for normality  - if the tests used are obscure, why did the authors used them and have they included a reference.  - if point estimates and measures of variability were presented for the primary outcome  - if subgroups were analysed appropriately  - if potential confounders were identified and taken into account in the analysis  - if there was any adjustment made for multiple testing  - if missing data was handled appropriately |
| **Comments** | | | *Add any other relevant comments, including if this is likely to influence the results of the study* | |
| **What is the overall risk of bias?** | | | Low  Moderate  High  Insufficient information | *Low* ***-*** *All of the criteria have been fulfilled or where criteria have not been fulfilled it is very unlikely the conclusions of the study would be affected.*  *Moderate - Some of the criteria have been fulfilled and those criteria that have not been fulfilled may affect the conclusions of the study.*  *High - Few or no criteria fulfilled or the conclusions of the study are likely or very likely to be affected.*  *Insufficient information – not enough information provided on methodological quality to be able to determine risk of bias.* |

**Cited in full as:** Monash Centre for Health Research and Implementation (MCHRI) Evidence Synthesis Program template for critical appraisal of a cohort study (2014), MCHRI – Monash University and Monash Health, Melbourne, Australia (*adapted from* Critical Appraisal Templates (2010) Centre for Clinical Effectiveness, Southern Health, Melbourne, Australia AND Wells G, Shea B, O’Connell D, Peterson J, Welch V, Losos M, Tugwell P. The Newcastle–Ottawa Scale (NOS) for assessing the quality of nonrandomized studies in meta-analyses. In: Proceedings of the 3rd Symposium on Systematic Reviews Beyond the Basics: Improving Quality and Impact. Oxford; 2000. p. 3-5).
