# Supplementary material for: Prolactin in relation to gestational diabetes and metabolic risk in pregnancy and postpartum: A systematic review and meta-analysis
Source: Front Endocrinol (Lausanne). 2022 Dec 22;13:1069625. doi: 10.3389/fendo.2022.1069625 (PMC9813437; doi:10.3389/fendo.2022.1069625)

**Supplementary material 3: funnel plots**

**Funnel plot for meta-analysis of PRL levels in GDM vs non-GDM control women in early pregnancy (≤24 weeks) – 3 studies**

**
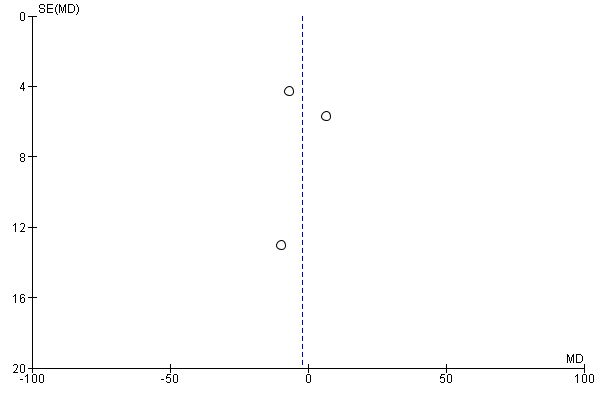
**

**Funnel plot for meta-analysis of PRL levels in GDM vs non-GDM control women in late pregnancy (>24 weeks) – 11 studies**


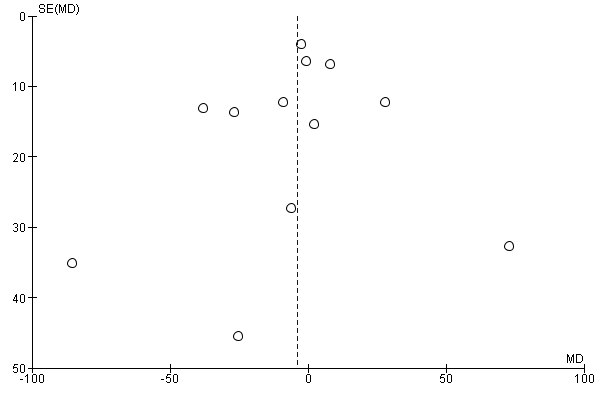

Supplement: Supplementary file 3 [file Table_3.docx]
